# Multidisciplinary group performance—measuring integration intensity in the context of the North West London Integrated Care Pilot

**DOI:** 10.5334/ijic.996

**Published:** 2013-02-15

**Authors:** Matthew Harris, Felix Greaves, Laura Gunn, Susan Patterson, Geva Greenfield, Josip Car, Azeem Majeed, Yannis Pappas

**Affiliations:** Department of Primary Care and Public Health, Imperial College London, 3^rd^ Floor, Reynolds Building, St. Dunstan’s Road, Hammersmith, W6 8RP, UK; Department of Primary Care and Public Health, Imperial College London, 3^rd^ Floor, Reynolds Building, St. Dunstan’s Road, Hammersmith, W6 8RP, UK; Department of Primary Care and Public Health, Imperial College London, 3^rd^ Floor, Reynolds Building, St. Dunstan’s Road, Hammersmith, W6 8RP, UK; Metro North Mental Health-Royal Brisbane and Women’s Hospital, Butterfield St., Herston, Queensland, Australia; Department of Primary Care and Public Health, Imperial College London, 3^rd^ Floor, Reynolds Building, St. Dunstan’s Road, Hammersmith, W6 8RP, UK; Department of Primary Care and Public Health, Imperial College London, 3^rd^ Floor, Reynolds Building, St. Dunstan’s Road, Hammersmith, W6 8RP, UK; Department of Primary Care and Public Health, Imperial College London, 3^rd^ Floor, Reynolds Building, St. Dunstan’s Road, Hammersmith, W6 8RP, UK; Department of Primary Care and Public Health, Imperial College London, 3^rd^ Floor, Reynolds Building, St. Dunstan’s Road, Hammersmith, W6 8RP, UK

**Keywords:** integrated care, multi-disciplinary groups, communication, health services

## Abstract

**Introduction:**

Multidisciplinary Group meetings (MDGs) are seen as key facilitators of integration, moving from individual to multi-disciplinary decision-making, and from a focus on individual patients to a focus on patient groups. We have developed a method for coding MDG transcripts to identify whether they are or are not vehicles for delivering the anticipated efficiency improvements across various providers and apply it to a test case in the North West London Integrated Care Pilot.

**Methods:**

We defined ‘integrating’ as the process within the MDG meeting that enables or promotes an improved collaboration, improved understanding, and improved awareness of self and others within the local healthcare economy such that efficiency improvements could be identified and action taken. Utterances within the MDGs are coded according to three distinct domains grounded in concepts from communication, group decision-making, and integrated care literatures—the Valence, the Focus, and the Level. Standardized weighted integrative intensity scores are calculated across ten time deciles in the Case Discussion providing a graphical representation of its integrative intensity.

**Results:**

Intra- and Inter-rater reliability of the coding scheme was very good as measured by the Prevalence and Bias-adjusted Kappa Score. Standardized Weighted Integrative Intensity graph mirrored closely the verbatim transcript and is a convenient representation of complex communication dynamics. Trend in integrative intensity can be calculated and the characteristics of the MDG can be pragmatically described.

**Conclusion:**

This is a novel and potentially useful method for researchers, managers and practitioners to better understand MDG dynamics and to identify whether participants are integrating. The degree to which participants use MDG meetings to develop an integrated way of working is likely to require management, leadership and shared values.

## Introduction

The ageing population and rising prevalence of people with chronic disease have led to a widely-recognised need for more coordinated, ‘joined up’ care [1] improving the patient journey and experience. As a result, multidisciplinary team working or inter-professional collaboration has become a key feature of health care across many health domains [2–5] and has been shown to bring about positive health benefits when compared with traditional non-multidisciplinary care. Although there is considerable heterogeneity in the way that multidisciplinary teams are deployed, in general there are some factors considered to be important enablers for effective team working. Successful multidisciplinary groups are likely to be the result of an interplay between systemic, organizational and interactional factors [6]. Much importance has been attributed to factors such as mutual respect, trust and willingness to collaborate as determinants of good interaction between different professional groups [6]. Professional support, leadership and communication [7] stand out as important elements, as does motivation, culture and professional power [8] but these are all variably defined concepts. It follows that there are empirical challenges in determining what constitutes an effective multidisciplinary group and how to measure that effectiveness, in part because it is a negotiated agreement between professionals of different expertise and contributions to patient care [5].

In an integrated care context, where professionals come together from entirely different organizational backgrounds as well as professional ones, multidisciplinary groups are also seen as key facilitators of integration. They are not only a forum to discuss and coordinate complex individual patient care but are also opportunities for participants to discuss, reflect on and develop strategies to change systems and processes within the local health economy. MDGs might be more or less effective at achieving this objective for a number of reasons—participants might not be prepared to engage with the thorny issues of organizational change; the meeting might replicate traditional, hierarchical power relationships; some individuals might dominate the conversations; or participants might exhibit antipathy towards one another. The degree to which participants use MDG meetings to develop an integrated way of working is likely to require management, leadership and shared values.

Inter-professional working in the context of integrated care has been conceptualized by Boon et al. [9] and more recently Willumsen et al. [10]. They describe a spectrum of collaboration types ranging from parallel (the least collaborative) to consultative, collaborative, coordinated, multidisciplinary, interdisciplinary and integrated (the most collaborative). Each of these is characterized by different forms of interaction. The consultative type for example, involves the seeking and giving of expert advice from one professional to another; however, an integrated type of collaboration is characterized by a non-hierarchical, seamless continuum of decision-making, guided by consensus and mutual respect [9]. There is little guidance on how to characterize the communication patterns within an MDG so that it can be located on this spectrum. It is reasonable to expect, for example, that an MDG exhibit elements of all these types in varying proportions, even during a single meeting. This presents an important empirical problem—how to characterize an MDG with respect to whether the participants are integrating or not.

In this study, we describe a method that we have developed to identify whether MDGs are or are not vehicles for delivering the anticipated efficiency improvements across the various providers. This is a novel tool and one that could support managers, researchers and practitioners to better understand the dynamics within an MDG and to find ways to improve its performance.

### Case study context

The North West London Integrated Care Pilot was developed to increase collaborative working between primary care teams and specialists as well as between health and social care services [11]. It brings together over one hundred general practices, two Acute Trusts, five Primary Care Trusts, two Mental Health Trusts, three Community Health Trusts, five Local Authorities and two voluntary sector organizations caring for a population of 500,000 people. Because of its size and ambitious aims, the ICP has been the subject of media interest [12–14]. The ICP involves three key interventions: a new IT tool, detailed care planning and Multidisciplinary Groups (MDGs). Sixteen MDGs meet around once per month in 10 different localities across North West London to elaborate carefully considered care plans for complex patients, in order to reduce unnecessary hospitalizations. MDG participants include GPs from a number of different practices, Allied Health Professionals (District nurses, Community Matrons and Social Workers) and Hospital Consultants from different Acute Trusts—an attempt to deliver virtual (i.e. not involving actual mergers) vertical and horizontal integration [1, 15]. In a typical MDG meeting, GPs take it in turns to each present one of their complex patients for discussion by the group and together explore ways to deliver more coordinated care around that patient. Secondary, but still important, objectives are for the MDGs to serve as forums for participants to exchange information and experience of the local health services, and thereby explore ways to improve health care services generally, not just for the patient subject of the Case Discussion, but for patients generally (IMB presentation June 2011). Early on in the ICP, the objective for health professionals to work in this more integrated way was clearly articulated:

“the MDGs are a *vehicle* for delivering productivity and efficiency improvements within and across the various providers … [participants should] move away from stereotypes, get to know each other, be reflective and responsive, increase the level of trust, coordination and collaboration across providers working together towards better patient care…..” (IMB Chair, IMB presentation August 2011 [authors’ emphasis added]).

## Conceptual framework

Our first challenge was to define ‘integrating’. We understood it to involve effective team-working and decision-making, and to involve good communication and the development of healthy inter-professional relationships, but not be constituted of only any one of these. Furthermore, we understood ‘integrating’, in the context of health services, to involve some sense of purpose towards improvement in or at least understanding of the local health economy, in order to identify potential improvements in the offer, access and design of services, beyond that pertaining to participants’ own organizations. We therefore defined ‘integrating’ as the process within the MDG meeting that enables or promotes an improved collaboration, improved understanding, and improved awareness of self and others within the local healthcare economy such that efficiency improvements could be identified and action taken.

The literature on effective team-working, decision-making, communication and inter-professional care provides some insight into the group dynamics that would support such a process [16]. Interaction Process Analysis (IPA) is one of the most widely applied measures of group decision-making and enables assessment of participants’ interaction style in terms of whether it is positive, constructive and supportive, or whether there is antipathy and tension. Bales’ model is grounded in the view that utterances that are solution-oriented, supportive, offering opinions and exhibiting empathy are much more likely to improve the dynamics between the participants [16–19]. Hence, they are an important first step toward integrated care. We draw also on Clark [20] who states that effective inter-professional working in multi-disciplinary teams requires individuals to be reflexive in their communication. This enables participants to transcend their own professional roles and routines, leading to learning and a more collaborative environment, also an important step towards integration [20]. Finally, Curry and Ham [1] note that health service integration can occur, on various levels—micro (the individual patient level), meso (groups and services) and macro (organizations). Professionals, services and organizations may work in an integrated way around the care of an individual patient, but this may not extend to other patients, or to general structures and processes. It follows, that in the context of a multi-disciplinary meeting, the content of the conversation is important because this influences the type of integration which can occur between participants. MDGs that focus exclusively on the specifics of an individual clinical case may integrate participants around that case but opportunities to address broader issues within the local health economy may be missed.

If MDGs are to be successful vehicles for delivering efficiency improvements across the various providers (a key objective of the ICP) then during Case Discussions we would expect discussion, in varying proportions, at all three levels—micro (e.g. care of the patient), meso (e.g. care of groups of patients) and macro (e.g. how organizations in the local health economy are working together); we would also expect participants to be reflexive in their practice and to be open to explore experiences of existing services; and finally we would expect participants to work in a supportive and collaborative way. Using Boon’s et al. [9] typology, a consultative MDG might score high in the giving of advice, low in reflection, and have an emphasis on the individual patient level. Conversely, we would expect an integrative MDG discussion to be high in reflection, to demonstrate positive, reinforcing interactions and to have a focus on the systems, local health economy and organizational environment.

Conceptualizing integrative intensity as a product of these three domains or axes (Table 1) i.e.

1. the **type** of interaction between the participants (we call this the Valence)
2. the degree of **reflexivity** (we call this the Focus) that participants are exhibiting
3. and the **content** of the conversations (we call this the Level),

we developed a coding scheme which can be applied to characterize communication within MDGs with respect to whether it is integrating. We took the Case Discussion as the unit of analysis and measured the verbal communication patterns of participants within the MDG as the mode of integration. In combination these domains afford us a ‘three-dimensional’ view of communication, and by extension the meeting, with respect to what we call its ‘integrative intensity’. We quantified the proportions of the MDG that were characterized in terms of the three conceptual domains and calculated a new measure of MDG performance that we call the standardized weighted integrative intensity score.

## Method

We tested the coding method in a pilot Case Discussion within the Integrated Care Pilot. The Case Discussion was audio recorded with participant consent and professionally transcribed verbatim. The transcript was checked for accuracy against the audio record and de-identified. Analysis involved several steps—first familiarity with transcript as a whole; next the transcript was segmented into units of meaning, phrases or sentences expressing a complete thought, identified linguistically based on intonation [21]. Dialogue was divided into sentences or phrases of approximately equal length or where significant shifts in meaning, object, or subject occurred within the dialogue as illustrated by the example below:

I’ve got one patient, a 93-year-old Caribbean islander./

He lives on his own in a one-bedroom flat/

His basic problem is that he’s also got vascular dementia…also hypoglycemia/

He has told me that he has got recurrent falls and a history of UTI./

His atrial fibrillation is under control.

A second researcher checked where the units of meaning began and ended and any disagreements were resolved through discussion and consensus. The two researchers independently coded the transcript according to the three domains (Level, Valence and Focus).

For the Valence domain, we draw on Bales’ [16] IPA however we aggregate his coding categories into a lower level of granularity—‘solidarity’, ‘tension release’, ‘agreement’, ‘giving suggestion’ and ‘giving opinion’ are coded into one category only (Solution). We aggregate ‘antagonism’, ‘shows tension’, ‘disagreement’, ‘asking for suggestion and ‘asking for opinion’ into one category only (Problem). We aggregate the remaining two categories, ‘asking for orientation’ and ‘giving orientation’, into the last category (Information). Thus, utterances are coded Solution if they express sympathy, demonstrations of affection, urging of unity or harmony, expressing cooperation or solidarity; praising, complementing or congratulating; any manifestation of cheerfulness, concurrence, and statements of moral obligation or affirmations of major beliefs or values. Utterances are coded Problem where they include a request for diagnosis or guidance in the problem-solving process, rejecting another person’s statement of information, opinion, or suggestion, embarrassment, negativity or being unfriendly. Utterances are coded Information if they report or request factual observations or experiences, descriptions or any routine request for repetition.

We use three codes within the Level domain: Individual, Collective and System. Utterances that describe the patient, the care of the patient, a health professional, whether directly or indirectly involved in that case, the patient’s lifestyle, home context, and members of the patient’s family (if referred to in the singular e.g. ‘his sister’) or other individuals are coded Individual. Collective utterances are those which describe any groups of people such as a patient’s family, patients and patient groups, the care of patients, groups and categories of health professionals, disciplines and specialties, protocols and guidelines for the care of patients in general or within specific clinical domains and specialties and any other group of individuals. Utterances at the Systems level describe services, organizations, clinics, clubs, hospitals, aspects of the provision of services, other organizations in the local health economy, whether mentioned by name or category.

For the Focus domain, utterances demonstrating reflexivity and inquiry into one’s own and others’ practices, opinions, and processes are coded as Abstract. Abstract utterances question the status quo and pursue a new order or level of consciousness in the speaker or in others. Common key words include ‘think’, ‘believe’, ‘sure’ and the interrogative words such as ‘why’, ‘how’ and ‘perhaps’. Concrete utterances are defined by the absence of reflexivity. They are specific, tangible, technical or procedural comments.

## Analytical strategy

Each utterance is coded with respect to each of these three domains—Valence (Problem, Information or Solution), Level (Individual, Collective or System) and Focus (Concrete or Abstract). For example, the utterance *“I think we should be trying to reduce hospital admissions”* would be coded Solution-Systems-Abstract. All utterances in the transcribed Case Discussion were coded first with respect to the Level, then with respect to the Valence and finally the Focus so that any bias to code preferentially towards one permutation of the three codes was minimized. We aggregate the three codes for each utterance into an Event Code, of which there are eighteen permutations, and we afford the coding scheme two premises—firstly, that utterances at the Individual Level offer less integrative potential than those at the Collective Level and less still than those coded at the Systems Level; utterances with a Problem Valence offer less integrative potential than those with an Information Valence and less still than those with a Solution Valence; and utterances with a Concrete Focus offer less integrative potential than those of an Abstract Focus.

Our second premise is that the eighteen permutations can be ordered into an ordinal scale of integrative intensity. An utterance coded Individual-Information-Concrete (for example *“The patient is 95 years old and has diabetes”*) is considered to have less integrative intensity than an utterance coded as System-Solution-Abstract (for example *“I think we should be trying to decrease hospital admissions”*). There are, however, six ways to arrange the ordinal scale depending on the order of the domains Valence, Level and Focus (Table 2). For example, the Event Code permutation System-Problem-Abstract can be ordered six different ways, affecting its location on the ordinal scale.

We allocated an evenly distributed weighting scale from 1 at the lowest end to 2 at the highest end, and calculated the average weight for the six variants of each of the eighteen permutations, which was then ordered. Table 3 shows this ordinal scale with some examples of utterances.

Using this coding scheme enables exploration of change in integrative intensity during Case Discussions and over time. Following Poole et al. [22], we divide the total utterances in a Case Discussion into ten equal deciles, corresponding approximately to ten equal time segments, count the number of utterances coded in each Event Code category within the time deciles, and adjust them to a standardized number of utterances overall. We then calculate a weighted mean for each Case Discussion decile reflecting how the utterances are coded and the average weighting for each event code permutation shown in Table 3. To this end, we generate a standardized weighted mean integrative intensity score of the discussion at each time decile and are able to describe its change throughout the duration of the case discussion and compare the characteristics of one case discussion directly with that of another. We are then able to explore whether conversations that begin, understandably, with the case presentation (i.e. a non-reflective exchange of information about the case) progress or not to ‘higher’ levels of abstraction, reflection and interaction, discussing issues shared and common to similar cases and furthermore to issues shared and common to all participants and their organizational domains. The gradient of the Standardized Integration Intensity graphs for the Case Discussions indicates whether, and the extent to which, participants were integrative during the case discussions.

## Results

### Inter-coder and intra-coder reliability

We assessed inter- (A1 v B1; A2 v B2) and intra-rater (A1v A2; B1 v B2) validity using Kappa scores—a statistical test that determines levels of agreement. The repeated coding was performed several weeks apart to avoid the possibility of code recall. Sim and Wright [23] have shown that chance agreement is affected by the number and prevalence of the codes and that Kappa scores should be adjusted for prevalence and interpreted in the context of the maximum Kappa obtainable. We therefore calculated a Prevalence and Bias Adjusted Kappa score (PABAK) [24] to ascertain the relative importance of both and their impact on the Kappa. We also calculated a Maximum Kappa for comparison so that we had a reference value against which the Kappa and PABAK could be compared (Table 4). All of our codes were independent, avoiding a potential Kappa inflation.

### Case discussion

**Figure 1.**
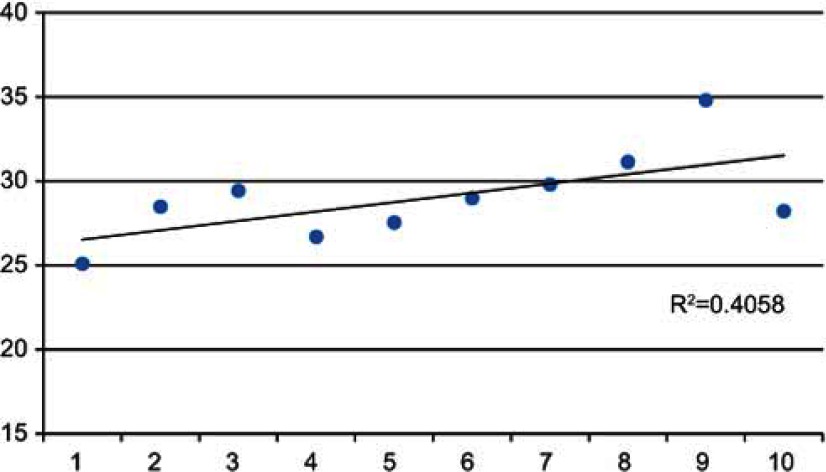
Standardised weighted mean integrative intensity scores at each decile during the Case Discussion.

The analysed case discussion involved six GPs all from different GP practices (one of whom was the presenting GP), two hospital consultants (one psychiatrist and one geriatrician) and three Allied Health Professionals (a Community Matron, a District Nurse and a Social Worker). The integrative intensity of the case discussion is shown across the ten time deciles (Figure 1). The discussion began in the first decile with the presentation of the case, measured as low integrative intensity by being non-reflective exchange of information at the individual patient level:

“I’ve got one patient, a 93-year-old Caribbean islander. He lives on his own in a one-bedroom flat. His basic problem is that he’s also got vascular dementia and recurring falls….” (Presenting GP)

In the second and third deciles participants reflected on whether he should continue to receive home physiotherapy or be put into a care home. Reflective comments, proposing solutions at a tertiary care level explain the rise in integrative intensity.

“He has had physiotherapy at St. Mary’s Hospital and from there they have referred to Westminster Rehabilitation Centre…..so I am just trying to avoid admission to secondary care unnecessarily.” (Presenting GP)

In the fourth decile, the presenting GP and Consultant returned the discussion to the patient’s clinical care, medication use and diagnostic tests that should be considered, which as a factual exchange of information led to a decrease in the integrative intensity score.

“Have you done the usual sort of falls work-up and everything?” (Consultant)

“Yes…he has got a full care plan, four times a day the carer is coming to look after him…” (Presenting GP)

In the fifth to ninth deciles, the Social Worker questioned whether a patient such as this should be sent into a care home and whether he would benefit instead from just being supported to be as independent as possible. This led to a reflective exchange on how the participants should be generally treating patients with such advanced age and complex medical conditions. They discussed and explored the services in the local region that could be drawn on to support the individual, and those in a similar situation to him. This shift in the conversation is represented in the gradually increasing integrative intensity scores through to the ninth decile.

“Then I ask you what does he want?.....We can’t just give up on people because they’re 95….Maybe our Reablement Service should be involved?.....He might be someone who could benefit from going somewhere like 60 Penfold Street” (Social Worker)

“How about a Falls Centre, so if he falls over that sets off a community alarm.” (Consultant)

“You need further discussion with Westminster Rehab service…that would get you through to KB she’s called, who’s our telecare officer in the City Council….” (Social Worker)

The decline in the tenth decile represents some general preamble to the next case discussion where participants were deciding who should present the case. The standardised weighted mean integrative intensity scores increased overall from the beginning of the case discussion to the end, indicating that there was some shift in integration from the baseline i.e. presentation of the case, through to consideration of broader issues around other types of patients, groups of patients, and the services available in the local health system. The case discussion started with discussion of a single case and ended with an improved awareness of the services available for this and other similar patients.

## Discussion

We have described a coding scheme that can be used to assess integration intensity of an MDG meeting, using the Case Discussion as a unit of analysis. We identify the preponderance of pre-determined conceptual domains in participants’ verbal communication that measure the extent to which participants were being supportive of each other (Valence), were speaking to individual, collective or systems issues (Level) and were being reflective (Focus). We understand these to be important enablers for MDGs to become vehicles for delivering efficiency improvements across the various providers—a key objective of the North West London Integrated Care Pilot. We were able to achieve good agreement between and within coders, indicating some reliability to the coding scheme.

We suggest that use of the approach can be of value to managers, researchers and participants alike. By identifying changes in integrative intensity during the Case Discussion we can draw some tentative conclusions about the collaborative characteristics of the meeting i.e. were they consultative or integrative [9]. The method could be used to support an objective quantification or characterization of the collaboration categories proposed by Boon et al. [9] and Willumsen et\xc2\xa0al. [10]. Quantification of qualitative data in this way allows for clear and transparent representations of conversation complexity and enables direct comparison between Case Discussions and between MDGs. The method could also be used to identify shifts in communication patterns over time as MDGs pass through their developmental stages, maturing and gaining confidence together to identify areas of improved collaboration outside of the meetings. This could be evidenced by an increasing gradient in the Case Discussions over time and might be used to identify which MDGs are failing to achieve this and why.

Although the standardized weighted mean integrative intensity scores appeared to mirror the content of the conversation, as is always the case with coding schemes, its validity is difficult to determine, particularly for a variously defined and understood concept such as integrated care [1, 25]. Other external measures of integration could be used to compare with our coding scheme [17], however, these also suffer from problems of validity and are often at a broader organizational level than that which could be correlated to those involved in the MDGs [26–27]. In the case of the NWL ICP the use (or not) of Out-of-Hospital Funds, a resource that can be used by the MDG participants to improve services in the community [11], might be a useful comparator. MDGs exhibiting highly integrative characteristics should be more likely to use the Out-of-Hospital funds. In future work, we will apply the coding scheme to several dozen Case Discussions across multiple MDG meetings and identify whether the results can be correlated to other external measures of integration. This is part of a broader, mixed methods evaluation of the ICP as a whole [28].

There are important methodological considerations with this approach to qualitative data. It has been argued that conceptual domains should be independent, that coding should be reliable, and the interactional structure of the utterances should be clear [29]. We found that the coding categories (Problem, Information, Solution, Individual, Collective, Systems, Concrete and Abstract) were sufficiently conceptually distinct so as to avoid any systematic overlap. Furthermore, we found that the coding rules were sufficiently detailed and clear so as to enable a good level of agreement between two independent coders. By grouping the coding categories into three distinct conceptual domains and coding each utterance three times this helped to improve agreement between the coders because the utterance content did not need to be constrained into just one coding category. Bales’ system has been criticized for being too prescriptive in that a single judgment needs to be made about potentially complex statements [19].

With this method, we are not assuming that our coding accurately represents that which the speaker him or herself intended to mean [30]. Our coding is simply a way to classify utterances against predetermined categories that are theoretically meaningful from the researcher’s perspective. Furthermore, we cannot comment on the intentionality behind MDG communication patterns [30]. If Case Discussions appeared to be integrative we are assuming that participants intended them to be so. Finally, we do not deliver an exhaustive conversation analysis, rather a pragmatic coding scheme of potential practical and empirical value. Coding schemes can be developed to tap into one or several theoretical concepts or communicative behaviors; utterances can be multidimensional even though by methodological convention they tend to be mutually exclusive with each behavior being classified into one and only one category [30]. There is always a trade-off between the number of categories and inter-observer reliability; our categories are relatively simple and require a low level of inferential coding judgment, which provides considerable operational convenience [30]. We recognize that we sacrifice some detail around the experience of the participants, in particular around power and dominance.

Multilevel content analysis of utterances has been used in other areas such as computer-supported learning [31] but we have not found empirical studies that use this methodology in the context of integrated care. Reviews of decision-making, team-working and communication in primary and community care reveal a paucity of empirical research examining real-time dynamics [32–34]. Frameworks to measure healthcare integration tend to draw on interviews and surveys, gathering perceptions, rather than actual dynamics [10]. Studies that code utterances, or units of meaning, as we have, explore MDGs within single organizations [35] not between organizations in a complex health economy.

In the healthcare context, integration is the process of bringing organizations and professionals together with the aim of improving outcomes for patients and service users through the delivery of integrated care [1, 25, 34]. MDGs afford opportunities to integrate care processes without the need to establish stand-alone organizational entities. If a Case Discussion exhibits a high or rapidly increasing integrative intensity score it still requires the skill and leadership of a good Chairperson to harness that conversation and identify actions to take forward that will improve the way these participants and their organizations work together outside of the meeting. Application of this coding scheme might reveal whether missed opportunities are due to this or the qualitative characteristics of the MDG conversation itself. Transparent assessment of the collaborative characteristics of the meeting can support a joint educative programme for all the participants, an important determinant of successful collaboration [6].

## Reviewers

**Gina Browne**, Professor, Health and Social Service Utilization Research Unit, Canada

**Nuria Toro**, Senior Researcher, Basque Foundation for Healthcare Innovation and Research, Spain

One anonymous reviewer.

## Figures and Tables

**Table 1.**
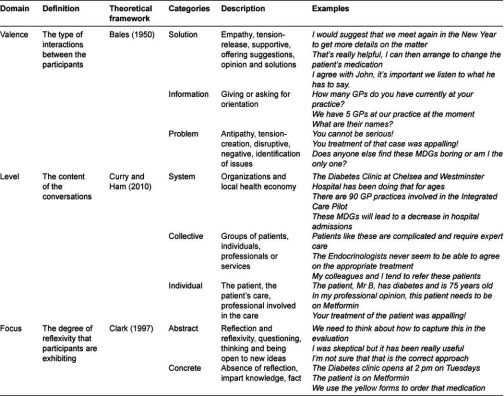
Conceptual framework for the measure of ‘integrative intensity’ with examples used in the coding method

**Table 2.**
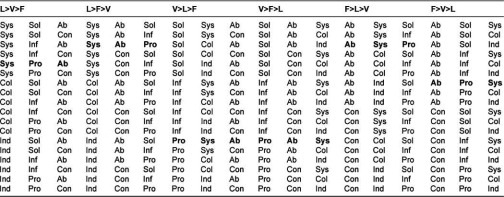
Six variations of the domains Level, Valence and Focus with their respective Event Code permutations

L=Level; V=Valence; F=Focus; Sys=Systems; Col=Collective; Ind=Individual; Sol=Solution; Inf=Information; Prob=Problem; Ab=Abstract; Con=Concrete.

**Table 3.**
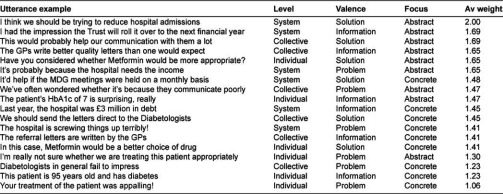
Average weighted ordinal scale with utterance examples

**Table 4.**
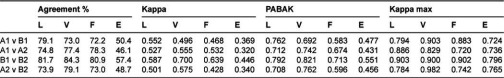
Agreement, Kappa, Prevalence and Bias adjusted Kappa and Kappa max

Comparison of the PABAK to the Kappa Max for each domain shows that the intra- and inter-rater agreement was very satisfactory. L=Level, V=Valence, F=Focus, E=Event Code (number of utterances = 155).
